# Caregivers Lack of Disclosure Skills Delays Disclosure to Children with Perinatal HIV in Resource-Limited Communities: Multicenter Qualitative Data from South Africa and Botswana

**DOI:** 10.1155/2016/9637587

**Published:** 2016-11-28

**Authors:** Sphiwe Madiba

**Affiliations:** School of Public Health, Department of Environmental and Occupational Heath, Sefako Makgatho Health Sciences University, Ga-Rankuwa, South Africa

## Abstract

To promote the appropriate implementation of procedures for health disclosure to children, it is important to understand the reasons why caregivers delay the disclosure of healthcare information to children. This paper explored the views of caregivers on what makes disclosure to children with perinatal acquired HIV (PAH) difficult and what could make disclosure in these cases easier. Data were collected using focus group interviews with caregivers who were purposely selected from a multicenter study conducted in Botswana and South Africa. Forty-seven nondisclosed caregivers of children between 5 and 18 years who were receiving ART were interviewed. Caregivers felt that children should be told of their HIV-positive status despite the fact that none had disclosed this information to the children. The caregivers reported lack of disclosure skills but believed they were primarily responsible for disclosure to children and required support from healthcare workers (HCWs) during the disclosure process. They believed that counseling on how to approach disclosure and training on when and how to disclose will make the disclosure process easier. HCWs have a crucial role to play in promoting disclosure to perinatally infected children. The development of appropriate disclosure guidelines and training for HCWs will facilitate disclosure to children.

## 1. Introduction

The introduction and expansion of pediatric antiretroviral treatment (ART) have increased the lifespan of children with perinatal acquired HIV (PAH). As more children with PAH are reaching adolescence, issues surrounding the disclosure of their HIV-positive status have become an important part of pediatric HIV care [1]. Disclosure of HIV status to children with PAH is beneficial and helps the child to gain a better understanding about the need to adhere to treatment and better self-care [2–4]. On the other hand, nondisclosure is one of the barriers to ART adherence among children and adolescents in resource-limited settings [5, 6]. Of concern in the area of public health are the low rates of disclosure to children and adolescents in resource-limited settings where the highest numbers of perinatally infected children reside [1, 5, 7–10]. It is also concerning that the reasons for these low rates of disclosure are not fully understood [4] because HIV status disclosure to children remains understudied in resource-limited settings [11].

The few studies that examine disclosure to perinatal infected children show various child and caregiver factors that are associated with disclosure to perinatally infected children [12]. These factors influence how and when caregivers decide to disclose to a child [11]. Although both child and caregiver related factors have been identified as barriers in disclosure to perinatally infected children, often studies that investigate disclosure to children focus on child related factors with the exception of caregiver related factors: The most cited child related factors for nondisclosure include perceived young age of the child, child's perceived ability to understand the meaning of HIV, and concerns that disclosure would have negative consequences for the child. There are also numerous child related fears that are cited as barriers to disclosure such as the fear that the child would tell others about their HIV status, fear of stigmatization, social rejection, and isolation, fear of the children's resentment, and fear that disclosure would hurt the child [2, 4, 5, 7–9, 13–15]. From the limited data on caregiver related barriers to disclosure, the most commonly cited include lack of HIV knowledge, lack of communication skills on HIV, lack of skills on how to conduct HIV disclosure, fear of answering questions related to the source of the HIV infection, unpreparedness for HIV related questions, lack of adequate knowledge about the benefits of disclosure, emotional unpreparedness to disclose, and fear of being judged and blamed by the adolescent [9, 10, 16, 17]. The identified caregiver related barriers suggest that disclosure is often delayed because caregivers lack the knowledge and skills to disclose to children. However, there is evidence that often caregivers of perinatally infected children require support from health professionals during the disclosure process or expect health professionals to disclosure on their behalf [2, 4, 9, 10, 14, 16, 18]. Therefore, to promote disclosure to children, it is important that the focus of investigations changes from exploring the social context of disclosure to the personal attributes and beliefs of the caregivers. The purpose of this paper is to answer the following questions: (1) what makes disclosure difficult for caregivers of perinatally infected children and (2) what would make disclosure easier. It is important that the challenges caregivers face regarding disclosure are explored and understood to facilitate the development of context-specific and locally relevant disclosure guidelines and procedures to promote disclosure to children with PAH [1, 10].

## 2. Methods

### 2.1. Study Design

This paper reports on the findings of two qualitative explorative studies conducted among primary caregivers of children with PAH receiving ART in a primary health care (PHC) clinic in Tshwane District, Gauteng province, South Africa, and the Infectious Disease Control Centre of Nyangabgwe Hospital, Francistown in Botswana. An exploratory design was selected because it provides an in-depth description of the study phenomenon [19]. Although both studies sought to explore caregivers' perceptions and motivation to disclose or withhold the status of children with PAH, they had slightly different objectives. The South Africa study focused on reasons for disclosure and nondisclosure, and the study in Botswana focused on perceptions about disclosure. Data analysis of the two studies yielded similar themes which formed the basis for reanalysis of the data.

### 2.2. Sampling

A purposive sample of caregivers of perinatal infected children was selected consecutively during routine visits to the clinics. Caregivers were selected for the study if they were over 18 years of age and had a child between 5 and 18 years who was receiving ART in the clinic. Both disclosed and nondisclosed caregivers were eligible to participate in both studies; however, this paper reports on data from nondisclosed primary caregivers. A primary caregiver was defined as an adult who was responsible for the care of the child in the home and for bringing the child to the clinic for his/her routine clinic follow-up.

### 2.3. Interviews

Focus group discussions (FGDs) were conducted by Master of Public Health (MPH) students and research assistants trained in qualitative methods using a semistructured interview guide with open-ended questions. The research assistants are trained field workers who support qualitative data collection for various projects in the School of Public Health. One-day training was conducted to explain the purpose of the study, the data collection tools, and the recruitment of study participants. Focus groups were selected as the method of choice for data collection because they are less threatening to many research participants and promote openness for participants to express detailed feelings, opinions, and attitudes about the topic under discussion [20]. The interviews for nondisclosed caregivers focused on the caregiver's personal experiences of caring for HIV-infected children, their perceptions of disclosure of the HIV status to the child, reasons for not disclosing, intentions regarding disclosure to children in their care, the preferred age for disclosure, the preferred person to disclose, and the nature of support they require during the process of disclosure.

All interviews were conducted in Setswana (a common language in both study settings) in a closed room away from the children to ensure privacy and avoid unintended disclosure to the children. The interview sessions were digitally recorded with permission from the caregivers. The caregivers were provided comprehensive information about the study objectives, confidentiality of the interview, and voluntary participation prior to the interviews. Six FGDs were conducted in the South African study with 49 caregivers and five FGDs were conducted in the Botswana study with 41 caregivers. In both studies, caregivers in FGDs were grouped as disclosed and nondisclosed, which made it possible to reanalyze the data from nondisclosed caregivers. From the South African study, there were 26 nondisclosed caregivers and 21 nondisclosed caregivers from the Botswana study, with a total of 47 nondisclosed caregivers. For both studies, the focus group interviews were guided by data saturation.

The interview format consisted of both open-ended questions and closed-ended questions to collect sociodemographic data such as caregiver age, gender, educational levels, marital status, employment status, HIV status of the caregiver, and relationship of caregiver to child. The caregiver also provided demographic information of their child such as gender, current age, age at first diagnosis, time since diagnosis, time since ART initiation, and child's orphan status.

### 2.4. Ethical Considerations

Ethical approval for both studies was granted by the Medunsa Research Ethics Committee (MREC) of the University of Limpopo. In addition, permission to conduct the South African study was granted by Tshwane District Health Research Committee and the Botswana study received approval from the Research and Ethics Committee of Botswana and Nyangabgwe Hospital. Participation was voluntary and caregivers were informed that they could withdraw from participation without negative consequences for them and the children under their care. Written informed consent was obtained from caregivers. Anonymity and confidentiality were ensured during data collection.

### 2.5. Data Analysis

Data from both studies were initially analyzed separately. Analysis began with the recorded interviews being transcribed verbatim in Setswana and translated into English by the MPH students under the guidance of the author. Thematic analysis was employed for the analysis of data and consisted of multiple readings of transcripts to understand the data and to identify emerging codes. Thematic analysis is a method for identifying, analyzing, and reporting patterns (themes) within data [21]. Data from both studies were then reanalyzed by the author to coincide with the aim and objectives of this article. Analysis began with repeated readings of the transcripts to familiarize with the data. This was followed by basic coding of the transcripts to generate initial codes from the data. From the initial list of codes, new themes and subthemes were identified to develop a codebook. The transcripts were then imported into NVivo version 10, qualitative data analysis software for application of codes and refining of the themes and subthemes. For presentation, data are grouped under themes and subthemes.

### 2.6. Trustworthiness

Credibility, transferability, dependability, and confirmability are strategies used to establish trustworthiness [22]. Triangulation, keeping an audit trail, making use of a good digital recorder, obtaining interview notes, transcribing the interviews verbatim, peer debriefing, and analyzing the data using NVivo qualitative software are strategies that were used to attain trustworthiness in the two studies. In the reanalysis of data, trustworthiness was enhanced by immersing in the data, triangulation of data sources, peer examination of data analysis processes with colleagues, employing the code-recode strategy, and maintaining an audit trail of all the data analyses activities [23].

## 3. Results

### 3.1. Description of Study Participants

The sample consisted of 47 caregivers who reported that they had not disclosed the HIV diagnosis to the child. The ages of the caregivers ranged from 27 to 73 years, almost all caregivers were female, and most of them were biological mothers and the rest were grandparents, aunts, and older siblings (Table 1).

The caregivers provided information about the child under their care. The age of the children ranged from 5 to 17 years, with most children receiving ART for 6–10 years (Table 2).

### 3.2. Themes

Four themes emerged from the data analysis: (1) perceptions of disclosure, (2) lack of disclosure skills, (3) making disclosure easier, and (4) preferred time of disclosure. Two subthemes explained making disclosure easier: (3.1) healthcare workers needed to support disclosure and (3.2) training and counselling on disclosure.

#### 3.2.1. Perceptions of the Importance of Disclosure

Even though none of the caregivers had disclosed, they felt that the children should be told about their HIV status. They perceived disclosure to children as a good practice and that the child has to know his/her status. Most reported that not telling the child that they have HIV was difficult and painful and they felt guilty about not disclosing the facts to their children.

*Eish…, it is really painful…, as a parent, you know the child's status while she does not know. It is painful…, when I am with the child and I know her diagnosis while she is in the dark, I ask myself when I am going to tell her. (mother of 12-year-old girl)*

*At first it was very difficult and painful especially when I look at him and recall what really happened to him. Sometimes I ask myself what will he think when he gets to know how HIV is transmitted, this really worries me a lot. (mother of 10-year-old boy)*

#### 3.2.2. Lack of Disclosure Skills

Although disclosure is often delayed because of child related reasons, caregivers also delay disclosure because of their own readiness. Caregivers were anxious about their lack of skills and ability to disclose to their child. Most of the caregivers felt that disclosure was complex and difficult and were concerned that they did not have the necessary skills to disclose.

*It is really difficult, sometimes I get worried and it pains me because I do not know what to say and which words to use. I am really afraid to disclose because I do not know how to start. (mother of an 11-year-old boy)*

*I want to disclose to my child, but the problem is that I do not know where I should start and what to say. I wish that the health care workers could tell me how to start. (mother of a 15-year-old boy)*

*I wish to disclose to my child but his condition worries me and I am not in the position to disclose because I do not know what to tell. (grandmother of a 9-year-old girl)*

*If one is not ready to disclose it become difficult to approach the child because sometime she will ask questions that you will not be in the position to answer. (mother of a 9-year-old girl)*

#### 3.2.3. Making Disclosure Easier

In response to what they thought could make disclosure easier to accomplish, caregivers reported that they needed assistance from HCWs during the process of disclosure.

Two main subthemes emerged from the analysis: the need for HCWs to support disclosure and training and counseling on disclosure.

*(1) Healthcare Workers to Support Disclosure*. Most of the caregivers desired support from HCWs to prepare them for disclosure. They hoped that HCWs support would help them answer any questions the children might ask. Most of the caregivers worried that the children would react negatively to disclosure and they hoped that support from HCWs would help them to deal with any negative emotional reactions to disclosure.

*I think I will need assistance from a social worker when I decide to disclose, because if I do it alone, the child won't understand anything. (grandmother of 6-year-old boy)*

*I want to disclose to her, but for me it's difficult…, maybe if I can have the support from the nurse or social worker, then I can disclose. (mother of a 7-year-old girl)*

*I think I will be brave and disclose to him, and when we come to the clinic they will be adding on what I've already told him, I want them to emphasize on what I've already told him. (aunt of a 14-year-old boy)*

The views expressed by some of the caregivers were that the HCWs and caregivers both have roles to play in the process of disclosure.

*I haven't disclosed to my child and I need someone who can do it on my behalf, I need maybe a nurse or doctor who can assist me in disclosing. I don't have the courage of telling my child, but when the disclosure takes place I want to be present as well. (mother of a 10-year-old boy)*

*I wish that the doctors and nurses disclose in my presence I take it he will understand better if told by them and that will also make it easier to disclose. (grandmother of a 13-year-old girl)*

*(2) Counseling and Training on Disclosure*. As already mentioned, most caregivers requested support from HCWs during the process of disclosure. Counseling was the main form of support HCWs could offer caregivers to assist them with disclosure. Caregivers also expressed the need for HCWs to train and educate them on when and how to disclose to the child and provide practical guidance on how to approach the child for disclosure.

*We need to be taught on how to approach our children, and how to start and what to say. The difficult part is that we do not know how to approach them and what to say to them about their status. (mother of a 10-year-old girl)*

*I believe we need to be empowered as caregivers on how to disclose by social workers, doctors, and nurses so that when it's time to disclose we will not feel guilty. (mother of the a 12-year-old boy)*

*Healthcare workers should provide counseling sessions to caregivers before disclosure to prepare parents psychological and emotionally so that discloser could be easier. Morning education should also continue because they are crucial to our children. (mother of an 11-year-old boy)*

*I think it the caregiver's responsibility to disclose but what is hindering us is knowledge of how to disclose. I wish that the hospitals should educate us how to disclose even at school our children should be educated about HIV. (mother of an 8-year-old girl)*

#### 3.2.4. Preferred Time of Disclosure

All caregivers stated that they had intentions to disclose to their children in the future. They preferred to disclose when they see that the child can understand the disease. For some of the caregivers, disclosure will occur when the child enters puberty.

*I think when I disclose to the child at the age of 15 years, the child won't tell people what she's suffering from because the child understands HIV better. (father of 13-year-old girl)*

*I think when the child is matured, grown to a certain stage where you can say it is the appropriate time to explain to the child, that is when I can disclose. (father of 6-year-old girl)*

*Before he starts dating girls, he should know his status. (grandmother of 12-year-old girl)*

While some caregivers would disclose when they feel that the child is prepared for disclosure, they explained some of the ways they will use to prepare the child as reflected in these excerpts.

*I will try to give some ideas time and again until she grasp and have knowledge of HIV. I think the best age is 7 years and I will start to give hints about HIV slowly so that by the time she reaches 10–12 years, she is fully disclosed and understand about her HIV status. (mother of a 6-year-old girl)*

*I kept her HIV a secret because I want to see if there is any knowledge about HIV. My wish is that if I see that there is a little bit of light and maturity I can disclose her HIV status. (grandmother of 9-year-old girl)*

*I will wait until she start to ask me question related to HIV. Then it will be the time to disclose because I take it that she will be having some insight. (father of 6-year-old girl)*

## 4. Discussion

Disclosure of HIV-positive status to children and adolescents has emerged as one of the greatest challenges facing primary caregivers and HCWs. The study found that caregivers felt that disclosure was important and that perinatally infected children should be told of their HIV-positive status despite the fact that none of the caregivers had disclosed this status to their children. The perceptions of caregivers about disclosure to perinatally infected children observed in this study are not different from previous studies [17, 24, 25]. While caregivers felt that disclosure was a difficult undertaking, they also felt guilty and sad about not telling the child that they have HIV. This finding is consistent with those reported among caregivers in Botswana and South Africa where they described nondisclosure as emotionally draining but also revealed that they regretted not disclosing and feared that withholding the child's status might be morally wrong [9, 25].

Disclosure is often delayed because of caregiver readiness to deal with the disease and disclosure to the child. The findings revealed that most caregivers delayed disclosure and were anxious about disclosing because they lacked skills on how or when to disclose to children. Not knowing how to disclose was reported as a barrier to disclose in earlier studies conducted in South Africa [9, 14]. Because caregivers lacked disclosure skills, they expressed the need for support from HCWs to prepare them for disclosure. The findings are in agreement with other studies highlighting that caregivers of HIV-infected children wished for substantial support for disclosure from HCWs [2, 4, 9, 10, 14, 16, 18]. In a study conducted with caregivers in Thailand, Siripong and colleagues [26] reported that caregivers in their study were unprepared and had limited understandings about disclosure to perinatally infected children. Similarly, studies conducted with HCWs confirmed that most caregivers lacked the skills and ability needed to disclose to their children [27–29].

Counseling was the main form of support caregivers requested from HCWs. Besides acquiring knowledge about HIV from counseling, Gyamfi and colleagues [3] argue that continuous counseling will make caregivers aware of the need to disclose which will subsequently increase early disclosure. Caregivers were also concerned that the child would react negatively to disclosure and felt that HCWs should train them on when and how to disclose to the child and provide guidance on how to approach the child for disclosure. Similar findings were reported in previous studies. Kiwanuka and colleagues [4] reported that caregivers in their study did not have the skills for handling the disclosure process or the skills to deal with anticipated adverse outcomes from disclosure. Since caregivers in this study and others lacked disclosure skills [4, 26], support from HCWs through training and consistent counseling is critical. Moreover, Abebe and Teferra recommend that disclosure counseling for caregivers to promote disclosure to children should be provided by trained HCWs [2]. Based on the expressed needs of caregivers in this study, the development of appropriate disclosure guidelines and training for HCWs will facilitate disclosure to children [1, 4].

Despite indicating that they needed support from HCWs during the disclosure process, generally, caregivers perceived themselves as primarily responsible for telling the child about the HIV diagnosis. This is consistent with previous studies conducted with caregivers [9, 25, 30] and healthcare providers [28, 29]. Moreover, caregivers felt that disclosure should be a shared responsibility of both the caregiver and the HCWs. While some caregivers felt that disclosure should be done at home, there were those who believed that disclosure should be done in health facilities in the presence of HCWs. However, the need for support is not meant to take away the caregivers' primary responsibility of disclosing; they only wanted to be supported by HCWs during the actual disclosure event. This was consistent with findings from the current research [3].

The findings support the suggestion that barriers to disclosure of HIV status to children are not mutually exclusive [10]. While the caregivers reported lack of disclosure skills as the main barrier to disclosure, concerns about the perceived young age of the child also deterred them from disclosing. The implication of the findings is that caregiver's knowledge and skill alone are not adequate to facilitate disclosure to children [12]. Similar to other studies, caregivers preferred to disclose between 12 and 15 years of age and believed that at the preferred age the child will be able to understand the meaning of being HIV-positive. The ability of the child to understand the HIV diagnosis and being able to keep it a secret was perceived by most caregivers as indicators of the child's readiness for disclosure [31]. In line with previous and current findings, caregivers based the timing of disclosure on the child's age and maturity [1, 4].

## 5. Conclusion

Caregivers delayed disclosure because of a lack of disclosure skills and they needed support from HCWs to prepare them for disclosure. They were concerned that because they lacked disclosure skills they would not be able to handle the child's negative reactions from disclosure. They therefore believed that counseling on how to approach disclosure and training on when and how to disclose will make disclosure easier. The findings further revealed that caregiver lack of skills and beliefs shaped and influenced how and when disclosure took place. Disclosure was delayed because of concerns about the perceived young age of children despite some being over the recommended age of disclosure.

Disclosure interventions should focus on developing the caregiver's disclosure skills and also provide information on the benefits of disclosure and the importance of early disclosure to HIV-infected children. These interventions will be achieved only if HCWs have disclosure guidelines appropriate to the local context. This will allow them to guide caregivers on how to best disclose to children. The involvement of HCWs in the disclosure process is essential in increasing the rates of disclosure to HIV-infected children.

## Figures and Tables

**Table 1:** Demographic characteristics of caregivers perinatal infected children.

	Number	Percent
*Age of caregiver*		
25–39 years	17	36
40–49 years	14	30
50–59 years	11	23
60–69 years	4	9
70–79	1	2
*Gender of caregiver*		
Male	6	13
Female	41	87
*Marital status*		
Single	24	51
Ever married	23	49
*Relation to the child*		
Biological mother	22	47
Biological father	3	6
Grandparents	10	21
Other relatives^*∗*^	12	26
*Employment status *		
Employed	22	47
Unemployed	19	40
Pensioner	5	11
Schooling	1	2
*Caregivers HIV status*		
Negative	14	30
Positive	30	64
Unknown	3	6
*Child orphan status*		
Orphan	16	34
Nonorphan	31	66

^*∗*^Other relatives includes older sibling, uncles, and aunts.

**Table 2:** Demographic and clinical variables of perinatal infected children.

	Number	Percent
*Current age*		
5–10 years	27	57
11–17 years	20	43
*Sex of child*		
Girl	26	55
Boy	23	45
*Age when ART was started*		
0–5 years	25	53
6–10 years	15	32
10–17 years	7	15
*Duration on ART*		
0–5 years	11	23
6–10 years	29	62
11 years	7	15
*Schooling*		
No	0	0
Yes	47	100
